# Isoalantolactone Enhances the Antimicrobial Activity of Penicillin G against *Staphylococcus aureus* by Inactivating β-Lactamase during Protein Translation

**DOI:** 10.3390/pathogens9030161

**Published:** 2020-02-26

**Authors:** Yonglin Zhou, Yan Guo, Zhongmei Wen, Xinxin Ci, Lining Xia, Yanling Wang, Xuming Deng, Jianfeng Wang

**Affiliations:** 1 Department of Respiratory Medicine, the First Hospital of Jilin University, Changchun 130021, China; zhouyl18@mails.jlu.edu.cn (Y.Z.); guoyan0334@126.com (Y.G.); wenzhongmei@aliyun.com (Z.W.); cixinxin@jlu.edu.cn (X.C.); 2 Key Laboratory of Zoonosis Research, Ministry of Education, College of Veterinary Medicine, Jilin University, Changchun 130062, China; wangyl01@vlandgroup.com; 3 College of Veterinary Medicine, Xinjiang Agricultural University, Urmuqi 830052, China; xulei18@mails.jlu.edu.cn

**Keywords:** β-Lactamase-positive *S. aureus*, Isoalantolactone, β-lactamase inhibitor, Penicillin G, Anti-infection

## Abstract

β-Lactamase-positive *Staphylococcus aureus* is one of the most prevalent multidrug-resistant pathogens worldwide and is associated with increasing threats to clinical therapeutics and public health. Here, we showed that isoalantolactone (IAL), in combination with penicillin G, exhibited significant synergism against 21 β-lactamase-positive *S. aureus* strains (including methicillin resistant *S. aureus*). An enzyme inhibition assay, a checkerboard minimum inhibitory concentration (MIC) assay, a growth curve assay, a time-killing assay, a RT-PCR assay and Circular Dichroism (CD) spectroscopy were performed on different β-lactamases or β-lactamase-positive *S. aureus* strains, in vitro, to confirm the mechanism of inhibition of β-lactamase and the synergistic effects of the combination of penicillin G and IAL. All the fractional inhibitory concentration (FIC) indices of penicillin G, in combination with IAL, against β-lactamase-positive *S. aureus,* were less than 0.5, and ranged from 0.10 ± 0.02 to 0.38 ± 0.17. The survival rate of *S. aureus*-infected mice increased significantly from 35.29% to 88.24% within 144 h following multiple compound therapy approaches. Unlike sulbactam, IAL inactivated β-lactamase during protein translation, and the therapeutic effect of combination therapy with IAL and penicillin G was equivalent to that of sulbactam with penicillin G. Collectively, our results indicated that IAL is a promising and leading drug that can be used to restore the antibacterial effect of β-lactam antibiotics such as penicillin G and to address the inevitable infection caused by β-lactamase-positive *S. aureus*.

## 1. Introduction

The emergence and spread of bacteria that are resistant to current antibiotics is an issue that has received worldwide attention in recent decades; these bacteria, such as β-lactam-resistant *Staphylococcus aureus*, carbapenem-resistant *Enterobacteriaceae* and several multidrug-resistant Gram-negative organisms, carry inactivated or non-inactivated enzymes [1,2]. *S. aureus* is a common nosocomial pathogen that causes many invasive diseases, such as endocarditis, pneumonia, septic arthritis, and osteomyelitis, especially in patients in the intensive care unit (ICU) using ventilators for prolonged periods or subjected to blood transfusion [3,4]. *S. aureus* rapidly evolved β-lactam resistance and other forms of strong resistance, posing a formidable challenge to antimicrobial therapy, with increasing reports of treatment failure and mortality in patients [5,6,7,8].

β-Lactams, such as penicillin derivatives and related β-lactam classes of cephalosporin, are a class of antibiotics that are frequently used worldwide as clinical therapeutics for the treatment of severe bacterial infections [9]. Most of the antibiotics in this category disrupt bacterial cell wall biosynthesis by preventing the formation of peptide cross-links between the adjacent polysaccharide chains in the peptidoglycan layer [10]. Unfortunately, several common clinical bacteria have been shown to exhibit high resistance to β-lactams via the production of β-lactamases, especially *S. aureus* [11]. Almost all *S. aureus* strains carry β-lactamases, although mutations in penicillin G-binding proteins are the most important mechanisms of resistance to β-lactams in methicillin-resistant *S. aureus* (MRSA) [3]. Compound therapy approaches are urgently needed to solve the problem of β-lactam resistant bacterial infections by targeting β-lactamase; however, the speed of development of new antibiotics has significantly declined in recent decades [12].

Previous research indicated the favorable effects of β-lactamase inhibitors, such as sulbactam, tazobactam and clavulanate. The strategy adopted is to use antibiotic adjuvants to preserve the efficacy of current antibiotics, leading to many opportunities and challenges for the development and application of enzyme inhibitors. However, this type of β-lactamase inhibitor showed poor inhibition of certain class D β-lactamases and nearly all class B β-lactamases. Additionally, these inhibitors act as antibiotics and have intrinsic antibacterial activity against some bacteria, such as *Acinetobacter* strains [13], which may induce the development of bacterial resistance [14]. Naturally, the identification of specific β-lactamase inhibitors, without antibacterial activity, is an alternative strategy to address the current problem of bacterial resistance [15].

The pharmacological activities of traditional Chinese medicinal herbs have received increasing interest, and significant achievements have been made in recent years [16]. Isoalantolactone (IAL), a eudesmanolide-type sesquiterpene flavonoid compound, has been isolated from *Atalantia buxifolia*, *Artemisia*, *Carpesium cernuum* and *Inula helenium*, and has been shown to have anti-inflammatory, anticancer, antiparasitic and antivirulence activities [17,18,19,20]. In addition, pharmacological research found that this natural compound is a promising candidate for the treatment of carcinoma in humans [21]. However, the effect of IAL on resistance-related enzymes has not yet been reported. To the best of our knowledge, this work is the first to show the inhibitory effect of IAL against β-lactamases and the potential treatment of β-lactamase-positive *S. aureus* infection with penicillin G.

## 2. Materials and Methods

### 2.1. Bacterial Strains and Chemicals

All isolated *S. aureus* strains were obtained from porcine samples collected in Shandong, China. *S. aureus* USA300, *S. aureus* USA400, MRSA 252, *S. aureus* ATCC29213 and ATCC25923 were purchased from American Type Culture Collection (ATCC). *S. aureus* 8325-4 was presented by Professor Timothy J. Foster [22]. IAL, penicillin G, meropenem, cefalotin and sulbactam sodium were purchased from the National Institute for the Control of Pharmaceutical and Biological Products (Beijing, China).

### 2.2. Enzyme Inhibition Assays

A modified β-lactamase inhibition assay was carried out in this study, as described previously [23]. In brief, 5 × 10^5^ CFUs/mL of mid-logarithmic-phase bacterial cells in TSB (trypticase soy broth) broth were supplemented with different concentrations of IAL and then shaken under aerobic conditions at 37 °C for 4 h, and the culture supernatant was collected by centrifugation. *E. coli* BL21 cells carrying a β-lactamase-1 or β-lactamase-7 expression vector [24] were cultured with isopropyl-β-D-thiogalactoside to induce the expression of β-lactamases and then ultrasonicated. Then, the supernatant was collected by centrifugation and 100 μL of this supernatant was mixed with 75 μL of phosphate-buffered saline (PBS) in a 96-well microtiter plate, followed by the addition of 25 μL of nitrocefin and incubation at 37 °C for 30 min. β-Lactamase activity was determined based on changes in color and absorbance. The OD_492_ value for each sample was determined in the 96-well plates using a microplate reader (Tecan Austria GmbH, Grödig, Austria) at room temperature. The supernatants from the untreated bacterial culture or the cultures of cells expressing recombinant β-lactamases (β-lactamase-1 and β-lactamase-7) were preincubated with various concentrations of IAL at 37 °C for 30 min. Then, the values were determined, as described above.

### 2.3. Real-Time RT-PCR Assay

The primers for *β-lactamases* listed in Table 1 were designed based on the *S. aureus* USA300 sequence and used to test all of the *S. aureus* strains by polymerase chain reaction (PCR) to determine the similarity among *S. aureus β-lactamases* (NCBI: GenBank: CP000730.1). Reverse transcriptase PCR (RT-PCR) assay was used to further confirm whether the expression of the *β-lactamase* genes was influenced by IAL. Briefly, USA300 was grown to the post-exponential growth phase in TSB at 37 °C with different concentrations of IAL (0-32 μg/mL). Total RNA was prepared as previously described [25]. RNA was reverse transcribed into cDNA using the EasyScript One-Step gDNA Removal and cDNA Synthesis SuperMix (TransGen, Beijing, China). The primer pairs used for RT-PCR are listed in Table 1 (bold font). The *S. aureus* 16S rRNA housekeeping gene was used as an internal control to quantify the relative expression levels of the samples.

### 2.4. Western Blot Assay

Western blot analysis was used to further validate the effect of IAL on β-lactamase expression, as described in our previous study [26]. Briefly, USA300 was cultured in TSB supplemented with various concentrations of IAL at 37 °C with constant shaking under aerobic conditions for 2–4 h. The culture supernatants were collected by centrifugation, mixed with sodium dodecyl sulfate (SDS) loading buffer, and placed at 100 °C for 5 min. The proteins were separated by 12% SDS polyacrylamide gel electrophoresis (PAGE) and transferred onto a polyvinylidene fluoride (PVDF) membrane. Then, the membrane was blocked with dried nonfat milk for 2 h at ambient temperature, incubated with an anti-β-lactamase mouse polyclonal antibody (diluted 1:500 in milk powder; mice were immunized with prokaryotically expressed and purified β-lactamases to obtain antibodies from serum) for 2 h and an HRP-conjugated secondary goat antimouse antiserum (diluted 1:2000 in milk powder) for an additional 1 h. Finally, the blots were tested with Amersham ECL Western Blot Detection Reagent (GE Healthcare, Chalfont St Giles, UK).

### 2.5. Secondary Structure Determination of β-Lactamases by CD 

The circular dichroism (CD) spectra of the β-lactamases were analyzed using a CD spectrophotometer (MOS-500; Bio-Logic, France), according to the method described in our previous study [27]. *E. coli* BL21 (DE3) PET-28a β-lactamases expression was induced by IPTG in the presence or absence of IAL (32 μg/mL). The enzymes were purified by Ni column chromatography for determination of the secondary structure (the concentration of each protein was adjusted to 0.5 mg/mL) using quartz cuvette cells with a 1 mm optical distance at ambient temperature. The scanning wavelengths ranged from 190 to 250 nm with a resolution of 0.2 nm and a bandwidth of 1 nm. A scanning rate of 50 nm/min with a 2 s response time was used three times for all the samples. The BeStSel web server was used to analyze the secondary structure measurements of the β-lactamases [28]. The normalized root-mean-square deviation (NRMSD) values of all the samples were less than 0.1.

### 2.6. Susceptibility Testing 

A modified broth microdilution checkerboard method (Luria-Bertani (LB) broth was used in this experiment) was performed to identify the synergy between IAL and various β-lactam antibiotics against the tested strains by following the Clinical and Laboratory Standards Institute (CLSI) guidelines [29]. Due to the poor stability of the aqueous solution of penicillin, the minimum inhibitory concentrations (MIC) values were determined based on visual turbidity after 16-18 h of incubation. The fractional inhibitory concentration (FIC) index value was used to evaluate the efficacies of the combinations. FIC index = (MIC value of IAL alone / MIC value of IAL in combination) + (MIC value of antibiotic alone / MIC value of antibiotic in combination). FIC index ≤ 0.5 was defined as synergistic, 0.5 < FIC index ≤ 1 was considered as additive, 1 < FIC index ≤ 2 was considered as no interaction, and FIC index > 2 was defined as antagonistic.

### 2.7. Growth Curves and Time-Killing Assays

A growth curve assay was used to evaluate whether IAL affects of the growth of the tested strains [30]. Briefly, *S. aureus* USA300, *S. aureus* USA400, *S. aureus* isolate ST1064-1 and *S. aureus* isolate ST3032 were cultured in a TSB broth medium at 37 °C with shaking at 200 rpm to an OD_600_ value of 0.3. The cultures were then evenly dispensed into four 50 mL Erlenmeyer flasks and IAL (or dimethyl sulfoxide used as a control) was added to the cultures at 0 μg/mL, 8 μg/mL, 32 μg/mL and 128 μg/mL. The bacteria were cultured at 37 °C with shaking at 200 rpm, and bacterial growth was monitored by measuring the OD_600_ at the indicated time points.

Similarly, time-killing assays were also used to evaluate the potential bactericidal effect of IAL in combination with penicillin G. The concentrations of penicillin G used on different *S. aureus* strains were all 2x MIC value of penicillin G. For this purpose, 5 × 10^5^ CFUs/mL of mid-logarithmic-phase bacterial cells in LB broth were supplemented with penicillin G (0.5–8 μg/mL), IAL (32 μg/mL), penicillin G (0.5–8 μg/mL) in combination with IAL (32 μg/mL), or DMSO (normal control). The culture was continued at 37 °C, and a portion of the culture was aspirated at 0, 1, 3, 5, 7 and 9 h for bacterial count determination. Serial 10-fold dilutions of the cultures were plated on antibiotic-free LB agar plates for incubation at 37 °C, and bacterial colonies were counted after 24 h.

### 2.8. Cytotoxicity Assays

The hemolytic activity and cytotoxicity of IAL were used to evaluate potential toxic side effects. Briefly, a sheep erythrocyte suspension was mixed with serial dilutions of IAL (0, 8, 16, 32, 64, 128, 256 μg/mL) in PBS in a final volume of 1 mL. Following incubation for 30 min at 37 °C, the sheep erythrocytes were pelleted by centrifugation. The hemolytic activities of the supernatants were detected by measuring the OD_600_ of the mixture. In addition, sheep erythrocytes treated with 0.1% Triton X-100 was used as a positive control and PBS was used as a negative control.

To determine the cytotoxicity of IAL, in brief, 2 × 10^4^ A549 cells (human lung epithelial cells, ATCC) were seeded in each well of a plate overnight and then incubated with various concentrations of IAL (0, 4, 8, 16, 32, 64, 128 μg/mL) for 8 h at 37 °C. The absorbance of the LDH released was measured at 492 nm in a microplate reader (Tecan, Austria) using KIT. Cells were treated with 0.02% Triton X-100 as the positive control, and the untreated cell sample was used as a negative control.

### 2.9. Mouse Model of Intranasal Lung Infection

Endonasal pulmonary infection with *S. aureus* USA300 was used to determine the combined therapeutic effect in vivo. Six to eight week-old C57BL/6J male mice (20 ± 2 g) were purchased from Liaoning Changsheng Biotechnology co., Ltd. The animal experiments were approved by and conducted in accordance with the guidelines from the Animal Care and Use Committee of Jilin University.

Overnight cultures of *S. aureus* USA300 were transferred into 100 mL of TSB at 37 °C to an OD_600_ of 0.5. The bacteria were washed three times and resuspended in PBS for lung infection. The mice were randomly assigned to five groups (17 mice per group for mortality studies), namely the control solvent treatment, IAL alone, penicillin G alone, IAL in combination with penicillin G and sulbactam in combination with penicillin G. The mice were mildly anesthetized by ether inhalation, and then, 30 μL of a *S. aureus* USA300 suspension containing 2 × 10^8^ CFU was instilled into the left nasal cavity for survival studies. The model mice were subcutaneously administered penicillin G (50 mg/kg), IAL (25 mg/kg), penicillin G (50 mg/kg) combined with IAL (25 mg/kg), penicillin G (50 mg/kg) in combination with sulbactam (25 mg/kg), or solvent, and then, the treatment was readministered every 8 h. The number of surviving/dead mice was recorded until day 6 post infection.

For bronchoalveolar lavage fluid experiments, bacterial load determination and histopathology, mice were inoculated with 1 × 10^8^ CFU of *S. aureus* USA300 using the same method. The mice (12 mice per group for collecting bronchoalveolar lavage fluid, and 5 mice per group for bacterial burden and histopathological observation) were euthanized with anesthesia, followed by cervical dislocation at 36 h post infection (bronchoalveolar lavage fluid was collected at 6 h, 12 h, 24 h and 36 h). Bronchoalveolar lavage fluid was collected by intratracheal instillation of 400 uL of sterile PBS. The lavage fluid was centrifuged at 1000 rpm for 5 min, and the supernatants were used for enzyme inhibition assays, as described above, and cytokine measurements using eBioscience’s Mouse ELISA Kits (10255 Science Center Dr., San Diego, CA92121, USA). The left lobes of the mice were photographed, weighed and homogenized for calculation of bacterial load via serial 10-fold dilution and plating. Paraffin sections of the lung tissues were prepared and visualized by light microscopy to analyze the histopathological relevance of staphylococcal pneumonia.

### 2.10. Statistical Analysis

SPSS version 19.0 (IBM Corp. Armonk, NY, USA) was used to analyze all the experimental data in this study, and data are presented as the mean ± standard deviation. Significant differences of the experimental data were analyzed by an independent Student’s t test, * indicates *p* < 0.05. ** indicates *p* < 0.01.

## 3. Results

### 3.1. Effect of IAL on β-Lactamase Activity

**Figure 1:**
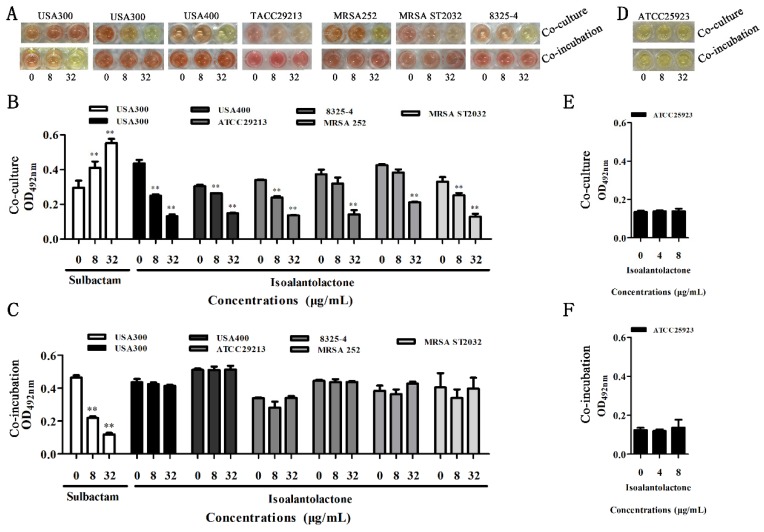
The activities of the β-lactamases of *S. aureus* strains were detected by enzyme inhibition assays after co-culture or co-incubation with IAL or sulbactam at concentrations of 0 µg/mL, 8/4 µg/mL and 32/8 µg/mL. The color changes of nitrocefin (**A**) were further detected by measuring the optimal OD of the mixture at 492 nm (**B**,**C**). The leftmost were treated with sulbactam, the rest were all treated with IAL. *S. aureus* ATCC 25923 was used as a negative control strain (**D**–**F**). The *S. aureus* USA300 treated with sulbactam was used as a positive control. ** indicates *p* < 0.01.

**Figure 2:**
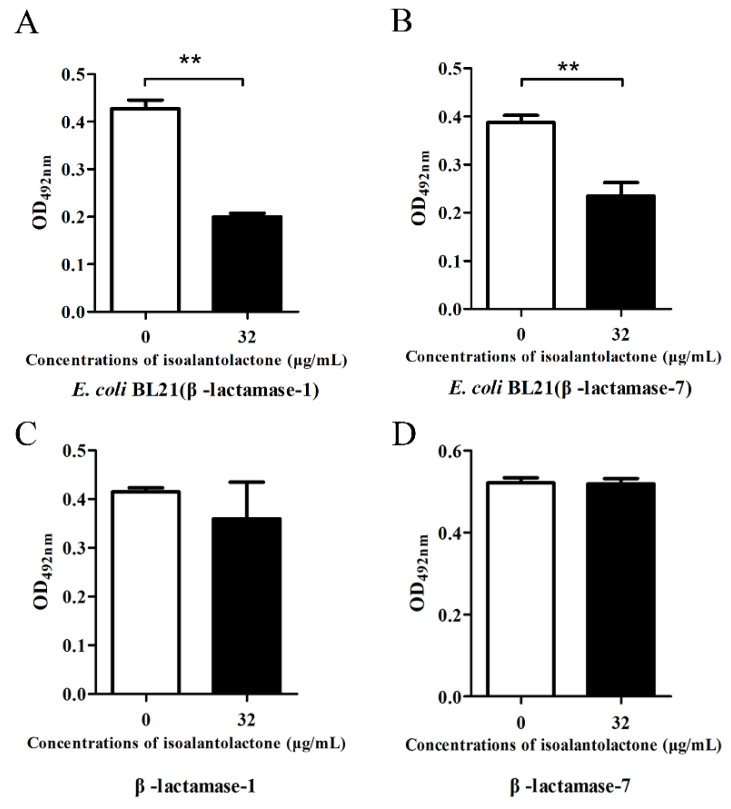
The activities of prokaryotically expressed β-lactamase-1 and β-lactamase-7 were detected by enzyme inhibition assays after co-culture (**A** and **B**) or co-incubation (**C** and **D**) with or without IAL. ** indicates *p* < 0.01.

We used modified inhibition assays to determine the effect of IAL on β-lactamase activity. Following co-culture with β-lactamase-positive *S. aureus*, IAL significantly inhibited β-lactamase activity in bacterial culture supernatant in a dose-dependent manner (Figure 1A,B). However, this inhibition was not observed in the sample (the supernatant from β-lactamase-positive *S. aureus*) co-incubated with IAL (Figure 1A,C). Interestingly, a marked increase in β-lactamase activity in the bacterial culture supernatant was detected upon co-culture with sulbactam, and sulbactam treatment led to the direct inhibition of β-lactamase activity in supernatants (Figure 1A–C). Notably, no inhibition was observed in the β-lactamase-negative *S. aureus* ATCC25923 culture supernatant, both co-cultured and co-incubated with IAL (Figure 1D–F). Consistent with the above results, the activities of β-lactamase-1 and β-lactamase-7 in lysed supernatants from *E. coli* BL21 co-cultured with IAL also significantly decreased (Figure 2A,B). Additionally, IAL incubation with the lysed supernatants did not lead to a visible reduction in β-lactamase activity (Figure 2C,D). Thus, our results suggested that, unlike sulbactam, IAL treatment might reduce β-lactamase production or bacterial viability. 

**Figure 3:**
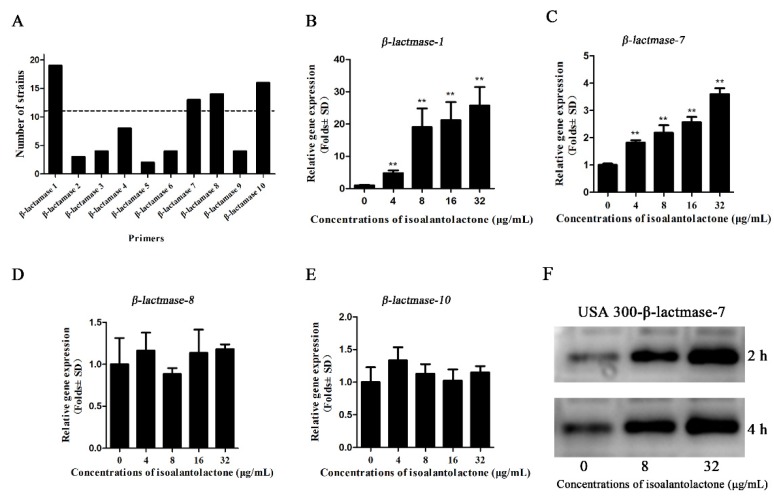
All of the *S. aureus* strains were tested by PCR with the primers designed for USA300 to determine the similarity among the β-lactamases of *S. aureus* (**A**). The dotted line indicated that 50% of the strains detected the β-lactamase. RT-PCR was used to further confirm the influence of IAL on the expression of *β-lactamase-1* (**B**), *β-lactamase-7* (**C**), *β-lactamase-8* (**D**), and *β-lactamase-10* (**E**). Western blot assays of β-lactamase-7 production (**F**). ** indicates *p* < 0.01.

**Figure 4:**
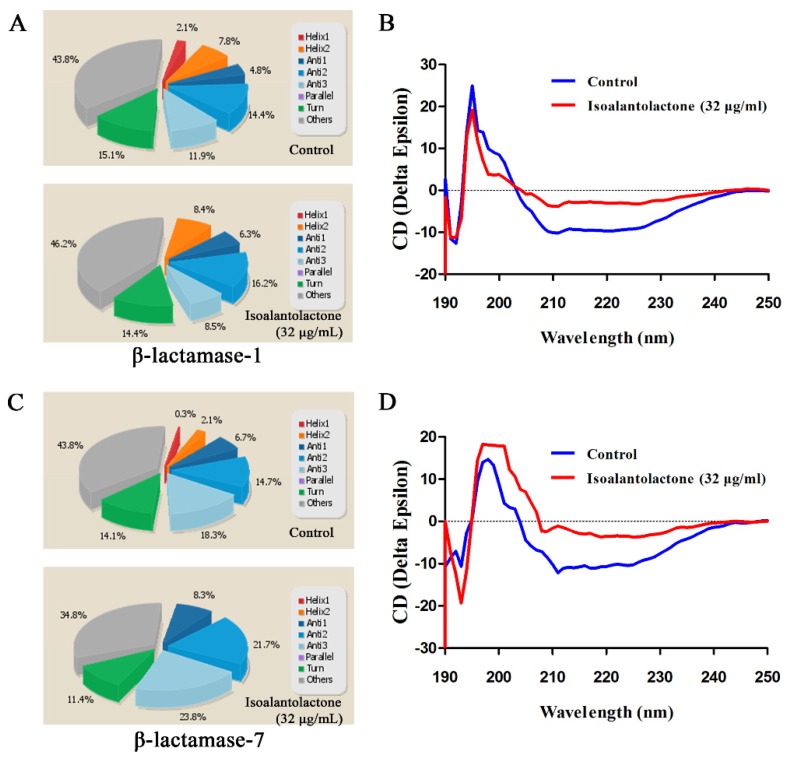
CD spectra of β-lactamase-1 (**A** and **B**) and β-lactamase-7 (**C** and **D**). The β-lactamases were treated with (red) or without (blue) IAL (**B** and **D**). The wavelength for CD spectroscopy was set as 190–250 nm.

Due to the importance and typicalness of the MRSA strain USA300, this strain was further used to determine whether IAL reduced the expression of β-lactamase by RT-PCR and Western blot assays. Based on the genome sequence of USA300, all of the genes encoding β-lactamases were detected in 21 *S. aureus* strains using a PCR-based assay. As shown in Figure 3A, four of the β-lactamases (β-lactamase 1, β-lactamase 7, β-lactamase 8 and β-lactamase 10) were found in more than 50% of the tested *S. aureus* strains and were chosen for further study. However, the transcriptional levels of the four β-lactamase genes were not inhibited following IAL treatment (Figure 3B–E). In contrast, the transcriptional levels of β-lactamase-1 and β-lactamase-7 were upregulated by IAL in a dose-dependent manner (Figure 3B,C). Consistent with this observation, the production of β-lactamase-7 in USA300 increased upon co-culture with IAL (Figure 3F). It was contradictory that the activity of β-lactamases was reduced and the production of β-lactamases was increased in the supernatants of USA300 co-cultured with IAL. These data suggest a loss of activity for IAL-induced hyperproduction of β-lactamase. In light of this hypothesis, the resulting secondary structures were determined via CD spectroscopy and are shown in Figure 4. IAL treatment caused an alteration in the β-lactamase conformation. The percentages of α-helix and turn conformations were both reduced in β-lactamase-1 (Figure 4A,B) and β-lactamase-7 (Figure 4C,D) after incubation with IAL. The β-sheet twist of the secondary structure in the BeStSel method was very important and caused a strong effect on the CD spectrumin. Thus, it was useful in dividing the antiparallel β-sheets into subgroups (anti1, anti2 and anti3). Our research found that the proportion of the total anti1 and anti2 increased in both of these β-lactamases.

### 3.2. Effective in Vitro Antimicrobial Activity of IAL in Combination with Penicillin G against β-lactamase-Positive S. aureus

A total of 21 *S. aureus* strains were examined for synergistic effects in this study, including MRSA and β-lactamase-negative *S. aureus* ATCC25923. It was performed to test for all bacteria, based on the results of the checkerboard MIC of *S. aureus* USA300 (Supplementary Figure S1). The results from the MIC between β-lactam antibiotics, in combination with IAL in β-lactamase-positive *S. aureus,* showed an MIC fold change ≥4 (FIC = 0.10 ± 0.02 - 0.38 ± 0.17) (Table 2). No synergistic effect was observed in β-lactamase-negative *S. aureus* ATCC25923 (FIC = 1.21 ± 0.07). This is mainly because ATCC25923 does not carry β-lactamase and ATCC25923 was highly sensitive to penicillin G (0.008 ± 0.000), so the synergistic effect could not be reflected. Additionally, a synergistic effect between sulbactam and penicillin G was also observed in β-lactamase-carrying *S. aureus* USA300 (FIC = 0.33 ± 0.04) (Table 2). Furthermore, the MICs of IAL for β-lactamase-positive *S. aureus* were all greater than 512 µg/mL, which is much higher (16-fold) than the concentrations used in the β-lactamase inhibitor screening and FIC determination assays.

**Figure 5:**
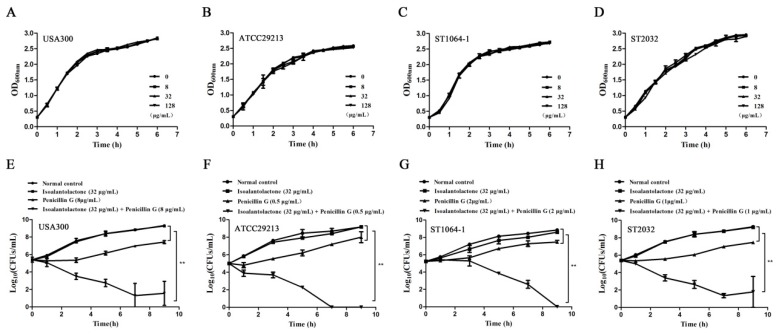
Growth curves for *S. aureus* USA300 (**A**), *S. aureus* ATCC29213 (**B**), *S. aureus* ST1064-1 (**C**) and *S. aureus* ST2032 (**D**) cultured in the presence of various concentrations of IAL (0–128 µg/mL). Time-killing curves of penicillin G, IAL, penicillin G + IAL, and a normal control (only medium) against *S. aureus* USA300 (**E**), *S. aureus* ATCC29213 (**F**), *S. aureus* ST1064-1 (**G**) and *S. aureus* ST2032 (**H**). Values represent the averages of three independent experiments. The penicillin G treatment compared to the normal control with short lines, and the combination compared to others with long lines, ** indicates *p* < 0.01.

Consistent with the MIC results, the growth of four tested β-lactamase-positive *S. aureus* strains was not affected in the presence of various concentrations (from 0 to 128 µg/mL) of IAL (Figure 5A–D). The potential bactericidal effect of IAL (32 µg/mL) combined with penicillin G (0.5, 1, 2 or 8 µg/mL) was also evaluated using time-kill assays. While no inhibition was observed for bacterial growth in the presence of IAL alone (32 µg/mL), the combination of IAL and penicillin G had an efficient bactericidal effect against these four tested β-lactamase-positive *S. aureus* strains (Figure 5E–H). Together, these results indicated that IAL treatment restored the antimicrobial activity of β-lactam antibiotics against β-lactamase-positive *S. aureus* without affecting bacterial viability.

### 3.3. Effective Antimicrobial Activity of IAL in Combination with Penicillin G in S. aureus USA300-Infected Mice

**Figure 6:**
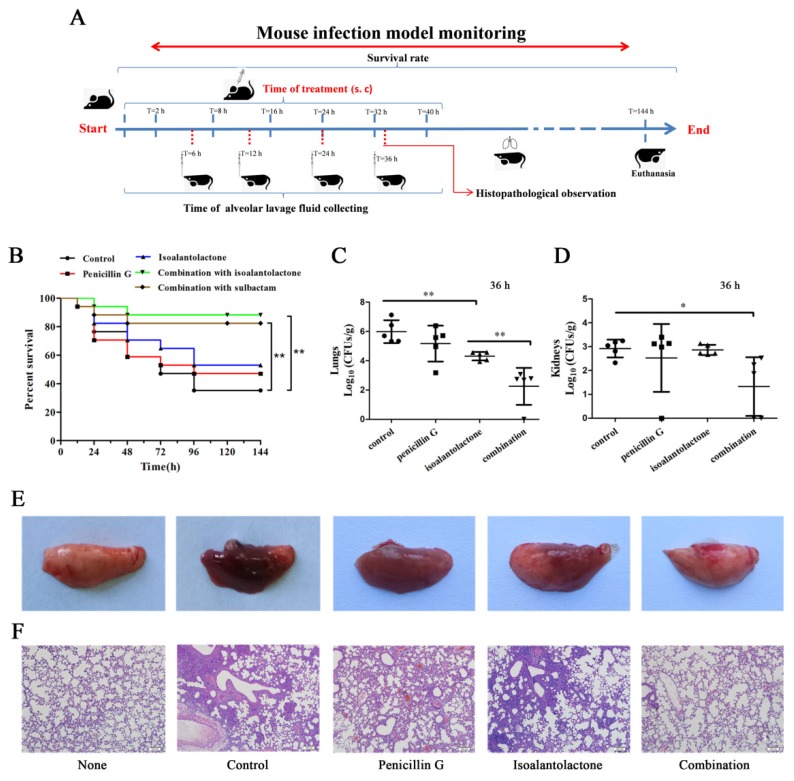
Effects of the IAL and penicillin G combination therapy in vivo. Mice were infected with *S. aureus* USA300 and then treated with IAL, penicillin G, IAL in combination with penicillin G, IAL in combination with sulbactam or control solvent. (**A**) Mouse infection model monitoring. (**B**) Survival plot of mice inoculated via intraperitoneal injection with *S. aureus* USA300. The bacterial burden in the lungs (**C**) and kidneys (**D**) was calculated. The gross pathological changes (**E**) and histopathology (**F**) of the lung tissue of mice were also assessed. * indicates *p* < 0.05. ** indicates *p* < 0.01.

We then attempted to confirm whether the synergy could be replicated in vivo (Figure 6A). Infected mice that received IAL in combination with penicillin G showed significant protection against *S. aureus* pneumonia for 144 h compared to other monotherapy groups (*p* < 0.001), suggesting that such a synergistic effect was also observed in vivo. Treatment with IAL alone also had a certain therapeutic effect. Importantly, 88.2% (14/17) of the mice treated with a combination therapy survived until the end of the experiment (Figure 6B). Additionally, this therapeutic and synergistic effect was equivalent to that of sulbactam combined with penicillin G. The bacterial load in the lungs was quantified to evaluate the influence of combination therapy on *S. aureus* survival. The combination of IAL and penicillin G resulted in a significant reduction in the bacterial load in the lung and kidneys compared to the other groups (Figure 6C,D).

**Figure 7:**
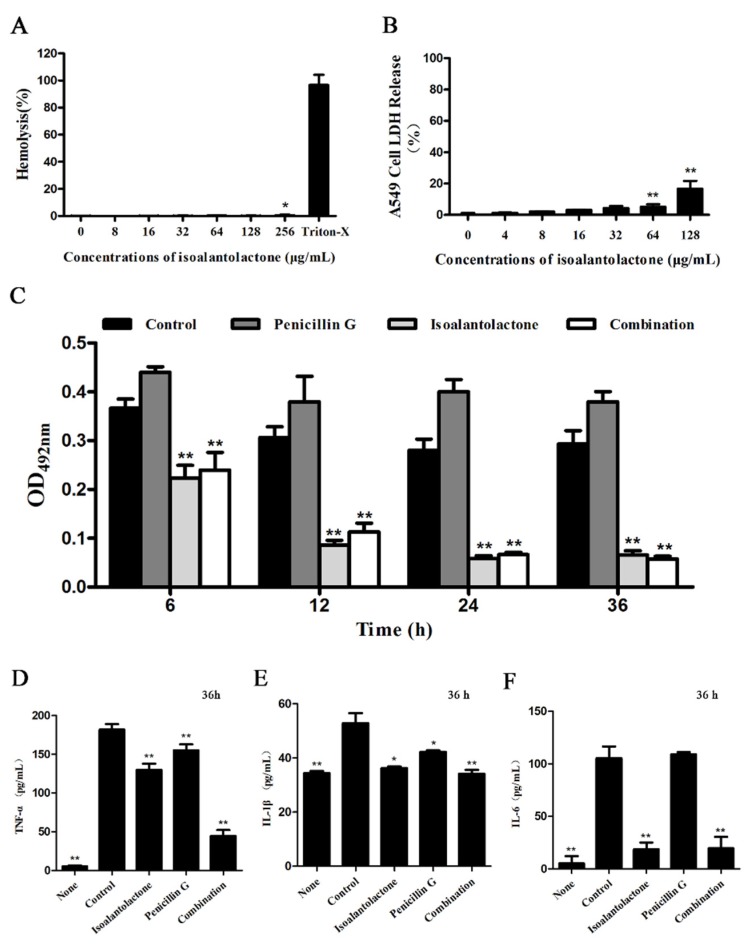
Hemolytic activity of IAL against red blood corpuscles (**A**). LDH release rates of A549 cells exposed to IAL solely (**B**). The activity of β-lactamase with different therapies in vivo was also detected by enzyme inhibition assays (**C**). TNF-α (**D**), IL-1β (**E**) and IL-6 (**F**) levels were assessed in the bronchoalveolar lavage fluid of mice (36 h after infection). * indicates *p* < 0.05. ** indicates *p* < 0.01.

Macroscopic inspection showed that the lungs of the mice in either the monotherapy groups or the control group were maroon and exhibited severe pulmonary tissue hyperemia and edema. Conversely, the lung tissues of mice, in the combination therapy groups, remained pink and fungous (Figure 6E), similar to those of uninfected mice. Severe tissue damage and accumulation of inflammatory cells were observed in the infected mice in the control group or monotherapy groups; however, such lesions were greatly alleviated in the mice in combination therapy groups (Figure 6F). In addition, the cytotoxicity of IAL against host cells was first determined using hemolysis and LDH assays. As shown in Figure 7A,B, IAL exhibits hardly any potential cytotoxicity at concentrations less than 64 µg/mL against sheep erythrocytes and A549 cells.

The potent inhibitory effect of IAL against β-lactamase activity in bronchoalveolar lavage fluid from infected mice was further determined. As expected, the activity of β-lactamases decreased by 50% or less after 12 h upon treatment with IAL or a combination; conversely, the activity of β-lactamases increased after 12 h upon treatment with penicillin G (Figure 7C). Furthermore, we detected the tumor necrosis factor-α (TNF-α), interleukin-1β (IL-1β) and interleukin-6 (IL-6) levels in the bronchoalveolar lavage fluid of infected mice. Either IAL alone or in combination with penicillinG resulted in a significant decrease in TNF-α, IL-1β and IL-6 levels in the fluid at 36 h post infection (Figure 7D–F). Taken together, the results show that the combination therapy with IAL and penicillin G provided systemic protection against *S. aureus* infection.

## 4. Discussion

Traditional β-lactamase inhibitors have certain antibacterial activities because these compounds are β-lactam compounds that irreversibly bind to β-lactamases, rendering the enzymes inactive, and thus protecting the penicillin [13,31]. Most of these β-lactamase inhibitors are highly effective against class A and class C β-lactamase activity, but are not significantly effective against the clinically relevant class D β-lactamases or class B metallo-β-lactamases [13,32,33]. However, the underlying mechanism of IAL inhibiting β-lactamases needs to be further studied. In addition, no in vitro inhibitory effect was observed against metallo-β-lactamases, such as NDM-1, as reported in our previous study [34]. In addition, a highly effective synergistic effect was observed for the combination of IAL, penicillin G and sulbactam, compared to that of the combination lacking sulbactam (data not shown). This finding suggested that no antagonism existed for the combination of IAL and sulbactam. Thus, IAL is a promising candidate for the treatment of infections caused by β-lactamase-producing bacteria when combined with β-lactam antibiotics. There are few studies on β-lactamases in *S. aureus* and other Gram-positive bacteria because of the emergence of serious clinical infections with Gram-negative bacteria carrying metallo-β-lactamases, such as NDM-1, IMP-1 and VIM-1 [35].

Our research found that the activity of β-lactamase-1 and β-lactamase-7 decreased, while transcriptional levels of β-lactamase-1 and β-lactamase-7 were upregulated after being co-cultured with IAL treatment. We were not sure whether the structure of β-lactamase changed. Additionally, our results only indicated that the secondary structure of β-lactamases (β-lactamase-1 and β-lactamase-7) were different when treated with IAL, as compared to the normal. The β-lactamase activity in bronchoalveolar lavage fluid increase in infected mice may have been due to the upregulation of β-lactamase. In addition, monitoring of the MIC showed that the synergistic effect of IAL and penicillin G decreased over time. The main reasons for this decrease could be as follows: i) the antibacterial mechanism of penicillin antibiotics do not target the cell wall quickly and do not completely kill bacteria; ii) MRSA has a high resistance to β-lactam antibiotics [36,37]; and iii) penicillin is easily inactivated at 37 °C [38,39].

Natural products are the main sources for drug discovery and development [40]. With further study on the detection techniques at the molecular level, traditional Chinese medicines are being used for the treatment of human ailments, such as malaria, for which adequate treatment and control methods have been developed since the discovery of artemisinin [41]. IAL is a sesquiterpene lactone compound that possesses various biological activities. Our previous study showed that IAL inhibited the production of α-toxin by *S. aureus* in vitro and protects mice from *S. aureus* pneumonia in vivo [20]. Taken together, the data from our research showed that IAL is potentially useful for the treatment of *S. aureus* pneumonia when used in combination with β-lactam antibiotics. In addition, IAL was administered by subcutaneous administration at a dose of 30 mg/kg, which had no macroscopic toxic effects, and no obvious adverse reactions were observed in C57/BL6 mice after intraperitoneal injection with IAL (20 mg/kg) [19,21].

In conclusion, our study showed that a combination of β-lactams and IAL might have been an alternative strategy for the treatment of infections caused by β-lactamase-mediated *S. aureus*. Therefore, IAL may be a candidate compound for future development of combination therapy against β-lactamase-positive bacteria.

## Figures and Tables

**Table 1:** The primers of *S. aureus* strain USA300 used for common pcr and RT-pcr.

Gene Names	Primer Names	Oligonucleotide Primer Sequence (5′–3′)
Metallo-beta-lactamase	β-lactamase-1-F	ccgggatccatgagccgcttgatacgcatgagtg
	β-lactamase-1-R	ccgctcgagttatattgtatatattggcgttgg
	**RT-β-lactamase-1-F**	gatgttgaatcgtttaacgtgtcacatgatgc
	**RT-β-lactamase-1-R**	ggataacgacacattctcaacatatcgacgtc
Possible beta-lactamase	β-lactamase-2-F	ccgggatccatgatcattgatcctatttgtga
	β-lactamase-2-R	ccgctcgagttatgtctttactatgaacag
Possible bifunctional beta-lactamase/ rhodanese domain protein	β-lactamase-3-F	cgcggatccatgttttttaaacagttttacga
	β-lactamase-3-R	ccgctcgagttattttaatgattctggaaaatc
Possible beta-lactamase / penicillin binding protein	β-lactamase-4-F	cgcggatccatgaaatttaataaagtaaaactagt
	β-lactamase-4-R	ccgctcgagttattgaacaataacacccttcg
AtsA/ElaC family / metallo-beta- lactamase	β-lactamase-5-F	cgcggatccatggaagttacattttttggaac
	β-lactamase-5-R	ccgctcgagttagattttaaaactatcaaaatct
Metallo-beta-lactamase	β-lactamase-6-F	cgcggatccatgaaacaattacatccaaatga
	β-lactamase-6-R	ccgctcgagttatttattgtttgattctttttgtt
Beta-lactamase	β-lactamase-7-F	cgcggatccatgagtttaataaagaaaaagaataaag
	β-lactamase-7-R	ccgctcgagttaaatttcagaaattactggaataat
	**RT-β-lactamase-7-F**	cacacggacatgagcacgcgattggtgc
	**RT-β-lactamase-7-R**	tatgaagtgtgaatacaaacacctaaac
Metallo-beta-lactamase	β-lactamase-8-F	cgcggatccatgaggatttcaagcttaactt
	β-lactamase-8-R	ccgctcgagttaaccgtgtaaaaatggattt
	**RT-β-lactamase-8-F**	cctggtgaatgtccaggtgtgtgtaac
	**RT-β-lactamase-8-R**	gatatagttgatcgattcgatgtcccgg
Possible metallo-beta-lactamase	β-lactamase-9-F	cgcggatccatgactaatcaatttaaaaata
	β-lactamase-9-R	ccgctcgagttagctattctcgccctcacgt
Possible metallo-beta-lactamase	β-lactamase-10-F	cgcggatccatgaagttatcatttcatggt
	β-lactamase-10-R	ccgctcgagttaaaactgaacagattcacctgg
	**RT-β-lactamase-10-F**	cagacctgtatcaccagtatgataaattg
	**RT-β-lactamase-10-R**	ggctgactatctttcttcatatcacggtg
*S. aureus* 16S ribosomal RNA	**RT-16S rRNA-F**	tgggatttgcttgacctcgcgg
	**RT-16S rRNA-R**	gggggacaaagtgacaggtggt

**Table 2:** MIC and FIC values of the penicillin G in combination with IAL or sulbactam for each of the tested bacterial strains.

Species	Source	β-lactamase Confirm	MIC (μg/mL)	Antibiotics	MIC (μg/mL)		FIC Index
			IAL		Alone	Combination	
				Penicillin G	53.33 ± 18.48	5.33 ± 2.31	0.19 ± 0.00
*S. aureus* USA300	American Type Culture Collection (ATCC)BAA-1717	+	≥512	Meropenem	1.33 ± 0.58	0.27 ± 0.07	0.25 ± 0.00
				Cefalotin	2.67 ± 1.15	0.50 ± 0.00	0.27 ± 0.07
*S. aureus* USA400	American Type Culture Collection (ATCC)	+	≥512	Penicillin G	3.33 ± 1.15	0.50 ± 0.00	0.23 ± 0.07
*S. aureus* ATCC29213	American Type Culture Collection (ATCC)	+	≥512	Penicillin G	2.00 ± 0.00	0.25 ± 0.00	0.19 ± 0.00
*S. aureus* 252	American Type Culture Collection (ATCC)	+	≥512	Penicillin G	32.00 ± 0.00	10.67 ± 4.62	0.38 ± 0.17
*S. aureus* 8325-4	presented by Professor Timothy J. Foster	+	>512	Penicillin G	0.50 ± 0.00	0.063 ± 0.00	0.19 ± 0.00
*S. aureus* ATCC25923	American Type Culture Collection (ATCC)	—	21.33 ± 9.24	Penicillin G	0.008 ± 0.000	0.008 ± 0.000	1.21 ± 0.07
MRSA ST1010	Obtained from the First Hospital of Jilin University in Jilin, China	+	≥512	Penicillin G	42.67 ± 18.48	8.00 ± 0.00	0.27 ± 0.07
MRSA ST1015-1	Obtained from porcine samples collected in Shandong, China	+	≥512	Penicillin G	64.00 ± 0.00	16.00 ± 0.00	0.31 ± 0.00
MRSA ST1015-2	Obtained from porcine samples collected in Shandong, China	+	≥512	Penicillin G	64.00 ± 0.00	13.33 ± 4.62	0.27 ± 0.07
MRSA ST1016-1	Obtained from porcine samples collected in Shandong, China	+	≥512	Penicillin G	16.00 ± 0.00	4.00 ± 0.00	0.31 ± 0.00
MRSA ST1053	Obtained from porcine samples collected in Shandong, China	+	≥512	Penicillin G	13.33 ± 4.62	2.33 ± 1.53	0.23 ± 0.07
MRSA ST1060	Obtained from porcine samples collected in Shandong, China	+	≥512	Penicillin G	32.00 ± 0.00	8.00 ± 0.00	0.31 ± 0.00
MRSA ST1061-1	Obtained from porcine samples collected in Shandong, China	+	≥512	Penicillin G	21.33 ± 9.24	5.33 ± 2.31	0.31 ± 0.00
MRSA ST1061-2	Obtained from porcine samples collected in Shandong, China	+	≥512	Penicillin G	8.00 ± 0.00	0.83 ± 0.29	0.17 ± 0.04
MRSA ST1064-1	Obtained from porcine samples collected in Shandong, China	+	≥512	Penicillin G	21.33 ± 9.24	1.83 ± 1.89	0.14 ± 0.05
MRSA ST1064-2	Obtained from porcine samples collected in Shandong, China	+	≥512	Penicillin G	34.67 ± 28.10	8.67 ± 7.02	0.31 ± 0.00
MRSA ST1067-1	Obtained from porcine samples collected in Shandong, China	+	≥512	Penicillin G	42.67 ± 18.48	5.33 ± 2.31	0.19 ± 0.00
MRSA ST1068-1	Obtained from porcine samples collected in Shandong, China	+	≥512	Penicillin G	32.00 ± 0.00	4.00 ± 3.46	0.19 ± 0.11
MRSA ST2022	Obtained from porcine samples collected in Shandong, China	+	≥512	Penicillin G	16.00 ± 13.86	2.00 ± 1.73	0.19 ± 0.00
MRSA ST2064	Obtained from porcine samples collected in Shandong, China	+	≥512	Penicillin G	106.7 ± 36.95	4.00 ± 0.00	0.10 ± 0.02
MRSA ST2032	Obtained from porcine samples collected in Shandong, China	+	≥512	Penicillin G	6.67 ± 2.31	0.67 ± 0.29	0.19 ± 0.06
			**Sulbactam**				
*S. aureus* USA300	American Type Culture Collection (ATCC)BAA-1717	+	128.00 ± 0.00	Penicillin G	53.33 ± 18.48	3.33 ± 0.33	0.313 ± 0.00

All MICs were determined in triplicate. The concentrations of IAL in combination therapy were 32 μg/mL to all bacteria, except for ATCC29213 were 4 μg/mL, the concentrations of sulbactam in combination therapy were 32 μg/mL to USA300. The data are presented as the mean ± standard deviation.

## Supplementary Materials

The following are available online at https://www.mdpi.com/2076-0817/9/3/161/s1, Figure S1: Checkerboard MIC analysis showing the combined effect of IAL and penicillin G against MRSA strain USA300.

## References

[1] Rolinson G.N. (1961). Celbenin—Resistant Staphylococci. Br. Med. J..

[2] Wang T.Z., Kodiyanplakkal R.P.L., Calfee D.P. (2019). Antimicrobial resistance in nephrology. Nat. Rev. Nephrol..

[3] Turner N.A., Sharma-Kuinkel B.K., Maskarinec S.A., Eichenberger E.M., Shah P.P., Carugati M., Holland T.L., Fowler V.G. (2019). Methicillin-resistant Staphylococcus aureus: An overview of basic and clinical research. Nat. Rev. Microbiol..

[4] Niu H., Yee R., Cui P., Tian L., Zhang S., Shi W., Sullivan D., Zhu B., Zhang W., Zhang Y. (2017). Identification of agents active against methicillin-resistant staphylococcus aureus usa300 from a clinical compound library. Pathogens.

[5] Centers for Disease Control and Prevention (CDC) (2013). Antibiotic Resistance Threats in the United States.

[6] Wertheim H.F., Melles D.C., Vos M.C., van Leeuwen W., van Belkum A., Verbrugh H.A., Nouwen J.L. (2005). The role of nasal carriage in Staphylococcus aureus infections. Lancet Infect. Dis..

[7] Appelbaum P.C. (2006). The emergence of vancomycin-intermediate and vancomycin-resistant Staphylococcus aureus. Clin. Microbiol. Infect..

[8] Vestergaard M., Frees D., Ingmer H. (2019). Antibiotic Resistance and the MRSA Problem. Microbiol. Spectr..

[9] Bush K., Bradford P.A. (2016). β-Lactams and β-Lactamase Inhibitors: An Overview. Cold Spring Harbor Perspect. Med..

[10] Page M.I. (1999). The reactivity of beta-lactams, the mechanism of catalysis and the inhibition of beta-lactamases. Curr. Pharm. Des..

[11] Lee A.S., de Lencastre H., Garau J., Kluytmans J., Malhotra-Kumar S., Peschel A., Harbarth S. (2018). Methicillin-resistant Staphylococcus aureus. Nat. Rev. Dis. Prim..

[12] Quadri L.E. (2007). Strategic paradigm shifts in the antimicrobial drug discovery process of the 21st century. Infect. Disord. Drug Targ..

[13] Shapiro A.B. (2017). Kinetics of Sulbactam Hydrolysis by beta-Lactamases, and Kinetics of beta-Lactamase Inhibition by Sulbactam. Antimicrob. Agent. Chemother..

[14] Barrett T.C., Mok W.W.K., Murawski A.M., Brynildsen M.P. (2019). Enhanced antibiotic resistance development from fluoroquinolone persisters after a single exposure to antibiotic. Nat. Commun..

[15] Zhao W.H., Hu Z.Q., Hara Y., Shimamura T. (2002). Inhibition of penicillinase by epigallocatechin gallate resulting in restoration of antibacterial activity of penicillin against penicillinase-producing Staphylococcus aureus. Antimicrob. Agent. Chemother..

[16] Tu Y.Y. (2011). TU You-you won Lasker Debakey clinical medical research award--for her outstanding achievements in studies on artemisinin. Chin. J. Integr. Tradit. Western Med..

[17] Li Z., Qin B., Qi X., Mao J., Wu D. (2016). Isoalantolactone induces apoptosis in human breast cancer cells via ROS-mediated mitochondrial pathway and downregulation of SIRT1. Arch. Pharm. Res..

[18] Jin C., Zhang G., Zhang Y., Hua P., Song G., Sun M., Li X., Tong T., Li B., Zhang X. (2017). Isoalantolactone induces intrinsic apoptosis through p53 signaling pathway in human lung squamous carcinoma cells. PLoS ONE.

[19] He G., Zhang X., Chen Y., Chen J., Li L., Xie Y. (2017). Isoalantolactone inhibits LPS-induced inflammation via NF-kappaB inactivation in peritoneal macrophages and improves survival in sepsis. Biomed. Pharmacother..

[20] Qiu J., Luo M., Wang J., Dong J., Li H., Leng B., Zhang Q., Dai X., Zhang Y., Niu X. (2011). Isoalantolactone protects against Staphylococcus aureus pneumonia. Fems Microbiol. Lett..

[21] Ding Y.H., Song Y.D., Wu Y.X., He H.Q., Yu T.H., Hu Y.D., Zhang D., Jiang H., Yu K., Li X. (2019). Isoalantolactone suppresses LPS-induced inflammation by inhibiting TRAF6 ubiquitination and alleviates acute lung injury. Acta Pharmacol. Sin..

[22] Bartlett A.H., Foster T.J., Hayashida A., Park P.W. (2008). Alpha-toxin facilitates the generation of CXC chemokine gradients and stimulates neutrophil homing in Staphylococcus aureus pneumonia. J. Infect. Dis..

[23] Liao D., Yang S., Wang J., Zhang J., Hong B., Wu F., Lei X. (2016). Total Synthesis and Structural Reassignment of Aspergillomarasmine, A. Angew. Chem..

[24] Teng Z., Guo Y., Liu X., Zhang J., Niu X., Yu Q., Deng X., Wang J. (2019). Theaflavin-3,3 -digallate increases the antibacterial activity of beta-lactam antibiotics by inhibiting metallo-beta-lactamase activity. J. Cell. Mol. Med..

[25] Qiu J., Feng H., Lu J., Xiang H., Wang D., Dong J., Wang J., Wang X., Liu J., Deng X. (2010). Eugenol reduces the expression of virulence-related exoproteins in Staphylococcus aureus. Appl. Environ. Microbiol..

[26] Zhou Y., Wang J., Guo Y., Liu X., Liu S., Niu X., Wang Y., Deng X. (2019). Discovery of a potential MCR-1 inhibitor that reverses polymyxin activity against clinical mcr-1-positive Enterobacteriaceae. J. Infect..

[27] Shen X., Niu X., Li G., Deng X., Wang J. (2018). Amentoflavone Ameliorates Streptococcus suis-Induced Infection In Vitro and In Vivo. Appl. Environ. Microbiol..

[28] Micsonai A., Wien F., Kernya L., Lee Y.H., Goto Y., Réfrégiers M., Kardos J. (2015). Accurate secondary structure prediction and fold recognition for circular dichroism spectroscopy. Proc. Natl. Acad. Sci. USA.

[29] Xu R., Polk R.E., Stencel L., Lowe D.K., Guharoy R., Duggal R.W., Wiest M., Putney K.S., Flint N.B., Flint N.B. (2012). Antibiogram compliance in University HealthSystem Consortium participating hospitals with Clinical and Laboratory Standards Institute guidelines. Am. J. Health-Syst. Pharm. AJHP.

[30] Li J., Dong J., Qiu J.Z., Wang J.F., Luo M.J., Li H.E., Leng B.-F., Ren W.-Z., Deng X.M. (2011). Peppermint oil decreases the production of virulence-associated exoproteins by Staphylococcus aureus. Molecules.

[31] Bou P.L.G. (2009). β-lactamase inhibitors: The story so far. Curr. Med. Chem..

[32] Sandanayaka V.P., Com S., Prashad A.S. (2002). Resistance to beta-lactam antibiotics: Structure and mechanism based design of beta-lactamase inhibitors. Curr. Med. Chem..

[33] Page M.G. (2000). β-Lactamase inhibitors. Drug Res. Updates.

[34] Liu S., Zhou Y., Niu X., Wang T., Li J., Liu Z., Wang J., Tang S., Wang Y., Deng X. (2018). Magnolol restores the activity of meropenem against NDM-1-producing Escherichia coli by inhibiting the activity of metallo-beta-lactamase. Cell Death Discov..

[35] Cain M.R. (2015). Design and Synthesis of Metallo-Β-Lactamase Inhibitors.

[36] Diep B.A., Gill S.R., Chang R.F., Phan T.H., Chen J.H., Davidson M.G., Lin F., Lin J., Carleton H.A., Mongodin E.F. (2006). Complete genome sequence of USA300, an epidemic clone of community-acquired meticillin-resistant Staphylococcus aureus. Lancet.

[37] Kuroda M., Ohta T., Uchiyama I., Baba T., Yuzawa H., Kobayashi I., Cui L., Oguchi A., Aoki K., Nagai Y. (2001). Whole genome sequencing of meticillin-resistant Staphylococcus aureus. Lancet.

[38] Brodersen R. (1947). Stability of penicillin G in aqueous solution as a function of hydrogen ion concentration and temperature. Acta Pharmacologica Et Toxicologica.

[39] Michnik A., Michalik K., Marcoin W. (2004). Influence of magnesium glutamate on stability of penicillin G aqueous solution. Int. J. Pharm..

[40] Newman D.J., Cragg G.M. (2016). Natural Products as Sources of New Drugs from 1981 to 2014. J. Nat. Prod..

[41] Nnankya M., Matthews G. (2016). Malaria Control: Better Health, Better Future 2016-Eradicate Malaria for Good. Outlooks Pest Manag..

