# Supplementary material for: Isoalantolactone Enhances the Antimicrobial Activity of Penicillin G against Staphylococcus aureus by Inactivating β-Lactamase during Protein Translation
Source: Pathogens. 2020 Feb 26;9(3):161. doi: 10.3390/pathogens9030161 (PMC7157633; doi:10.3390/pathogens9030161)
Supplement: Supplementary file 1 [file pathogens-09-00161-s001.pdf]

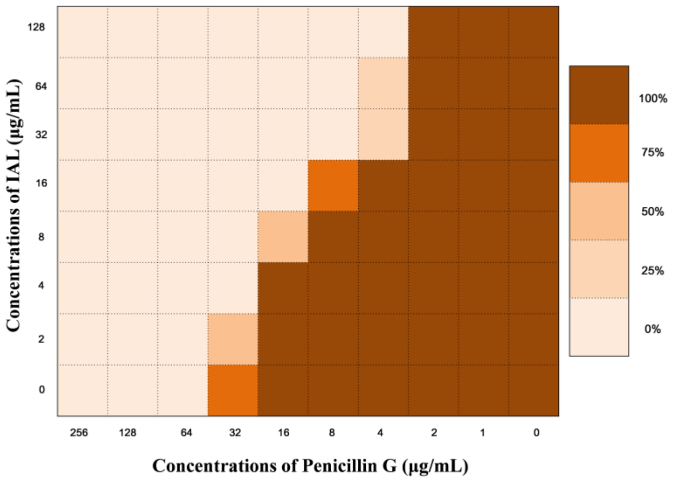

Supplementary Figure S1. Checkerboard MIC analysis showing the combined effect of IAL and penicillin G against MRSA strain USA300.
